# Genome-wide identification and analysis of WD40 proteins reveal that *NtTTG1* enhances drought tolerance in tobacco (*Nicotiana tabacum*)

**DOI:** 10.1186/s12864-024-10022-w

**Published:** 2024-02-02

**Authors:** Lijun Meng, Huan Su, Zechao Qu, Peng Lu, Jiemeng Tao, He Li, Jianfeng Zhang, Wei Zhang, Nan Liu, Peijian Cao, Jingjing Jin

**Affiliations:** 1 China Tobacco Gene Research Center, Zhengzhou Tobacco Research Institute of CNTC, Zhengzhou, 450001 China; 2 Beijing Life Science Academy, Beijing, 102200 China; 3 China National Tobacco Quality Supervision & Test Center, Zhengzhou, 450003 China

**Keywords:** Tobacco, WD40, Expression pattern, Drought

## Abstract

**Background:**

WD40 proteins, which are highly prevalent in eukaryotes, play important roles in plant development and stress responses. However, systematic identification and exploration of WD40 proteins in tobacco have not yet been conducted.

**Results:**

In this study, a total of 399 WD40 regulatory genes were identified in common tobacco (*Nicotiana tabacum*). Gene structure and motif analysis revealed structural and functional diversity among different clades of tobacco WD40 regulatory genes. The expansion of tobacco WD40 regulatory genes was mainly driven by segmental duplication and purifying selection. A potential regulatory network of NtWD40s suggested that NtWD40s might be regulated by miRNAs and transcription factors in various biological processes. Expression pattern analysis via transcriptome analysis and qRT-PCR revealed that many NtWD40s exhibited tissue-specific expression patterns and might be involved in various biotic and abiotic stresses. Furthermore, we have validated the critical role of *NtTTG1*, which was located in the nuclei of trichome cells, in enhancing the drought tolerance of tobacco plants.

**Conclusions:**

Our study provides comprehensive information to better understand the evolution of WD40 regulatory genes and their roles in different stress responses in tobacco.

**Supplementary Information:**

The online version contains supplementary material available at 10.1186/s12864-024-10022-w.

## Background

WD40 proteins, also known as WD40 domain-containing proteins, act as scaffolds for protein‒protein or protein‒DNA interactions, providing a platform for the recruitment of a variety of molecules to form functional complexes [1–3]. WD40 proteins are rarely found in prokaryotes but are highly prevalent in eukaryotes [4]. Each WD40 protein has several WD40 repeats, which contain 40–60 amino acid residue units as their main feature. Commonly, this unit contains glycine-histidine (GH) at the N-terminus and tryptophan-aspartate (Trp-Asp) at the C-terminus [5]. These conserved residues form a strong hydrogen bond network and stabilize the WD40 repeat fold [6, 7]. In addition, a single WD40 repeat contains a four-stranded antiparallel β–sheet [8]. Generally, WD40 protein motifs include 5–8 repeats, most often 7 repeats, which form a relatively stable β-propeller structure for various protein–protein interaction [9]. Therefore, investigating WD40 proteins is helpful for understanding their interactions with other proteins involved in various biological processes.

To date, WD40 proteins have been systematically explored in multiple plant species [10, 11], with 230 WD40s in *Arabidopsis thaliana* [11], 200 WD40s in rice (*Oryza sativa*) [12], 191 WD40s in cucumber (*Cucumis sativus*) [11], 315 WD40s in mango (*Mangifera indica* L.) [13], 579 WD40s in cotton (*Gossypium hirsutum*) [14], 743 WD40s in wheat (*Triticum aestivum*) [15], 231 WD40s in *Cucurbita maxim* [16]*,* 177 WD40s in *Sugar beet* [17], and 178 WD40s in potato (*Solanum tuberosum*) [18]. WD40 proteins play important roles in diverse biological processes, such as cell cycle regulation [19, 20], apoptosis[21], autophagy [22, 23], gene transcription [24], signal transduction [25, 26], histone modification [27, 28], and chromatin assembly [29, 30]. A WD40-repeat protein from the Recretohalophyte *Limonium bicolor* could enhance trichome formation and salt tolerance in *Arabidopsis* [31]. Germinating modulator of rice pollen (*GORI*), encoding the WD40 domain protein, is required for pollen tube germination and elongation in rice [32]. The TRANSPARENT TESTA GLABRA 1 (*TTG1*) protein from mango enhances root growth and abiotic tolerance in *Arabidopsis* [13]. Liu et al. have revealed that WD40-REPEAT 5a plays a role in drought stress tolerance by regulating nitric oxide accumulation in *Arabidopsis* [33]. Tian et al. have reported that the full-length allele TaWD40-4B.1C, which possesses nonsense nucleotide variation, enhancing the drought tolerance and grain yield of wheat plants under drought stress. TaWD40-4B.1C could interact with canonical catalases, promote their oligomerization and activities, and reduce H_2_O_2_ levels under drought stress. The knockdown of catalase genes erases the role of TaWD40-4B.1C in drought tolerance. The introgression of TaWD40-4B.1C enhances the drought tolerance of the cultivar harboring TaWD40-4B.1 [34]. The well-known MYB-bHLH-WD40 (MBW) complex plays a vital role in regulating the anthocyanin biosynthesis pathway in plants. For example, in barley (*Hordeum vulgare L.*), Ant13 encodes a WD40-type regulatory protein that is needed for the transcriptional activation of a set of structural genes encoding enzymes involved in flavonoid biosynthesis at the leaf sheath base and in grains [35]. For this cascading MBW regulatory model in rice, Sun et al. has reported that S1 (bHLH) acts as the master gene by activating the expression of C1 (MYB), after which C1 activates the expression of WA1 (WD40) during anthocyanin biosynthesis [36]. Yue et al. have demonstrated that the MBW complex associated with flavonoid metabolism in strawberry fruits is FaMYB5/FaMYB10-FaEGL3 (bHLH)-FaLWD1/FaLWD1-like (WD40). Moreover, it directly targets the promoters of flavonoid 3′-hydroxylase (F3'H), leucoanthocyanidin reductase (LAR) and H^+^-ATP (autoinhibited H^+^-ATPase isoform 10, AHA10), thereby promoting the accumulation of flavonoids [37]. Given the important roles of WD40 proteins in various plant biological processes, additional studies need to be performed.

Cultivated tobacco (*Nicotiana tabacum*), an allotetraploid plant, is an important model species for plant genetic research. TTG proteins, which are WD40 proteins, are essential regulators of plant trichome and seed development [38–40]. In tobacco, *NtTTG1* can regulate immune responses by recognizing a biotic elicitor and involving the transcription factor *NPR1*. In contrast, *NtTTG2* is an immune repressor that particularly suppresses the *NPR1* functional pathway to confer disease susceptibility. Transient expression of *TTG1* from *Raphanus sativus* results in an increase in anthocyanin accumulation in tobacco leaves [41]. However, WD40 studies in tobacco plants are still scarce. Moreover, genome-wide investigations of WD40s in tobacco have not been reported until now. In this study, a comprehensive analysis of WD40 proteins in tobacco was conducted, including phylogenetic relationship analysis, duplication analysis, functional element analysis, and expression pattern analysis in different tissues and in response to different biotic and abiotic treatments. Furthermore, the significant role of one WD40 protein, *NtTTG1*, in the drought response was validated. Our study provides comprehensive insights into the biological functions of WD40 proteins in tobacco and unveils important roles of WD40 proteins under different stress treatments.

## Results

### Genome-wide identification of WD40 regulatory genes in tobacco

**Fig. 1:**
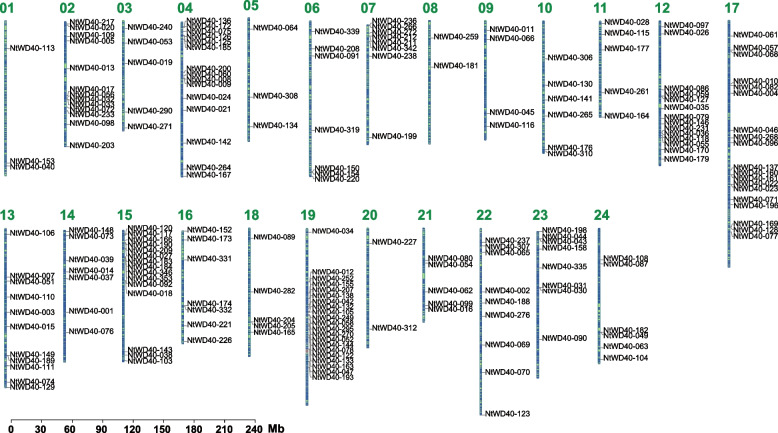
Chromosome distribution for 200 NtWD40 genes. The scale bar on the left indicates the length (Mb) of the tobacco chromosome. The chromosome number is shown on the left side of each chromosome

A total of 399 WD40 genes were identified based on comprehensive genome-wide analysis and were named NtWD40-001 to NtWD40-399 based on their location in the genome (Table S1). A total of 200 NtWD40 genes were unevenly distributed on 24 chromosomes, while the others were mapped onto scaffolds (Fig. 1). Chromosome 4 contained the highest number of WD40 genes (21), followed by chromosomes 17, 15, and 4 with 19, 15, and 15 genes, respectively. There were only 2 genes on chromosome 8. Subcellular localization prediction revealed that the tobacco WD40 regulatory genes were located mainly in the nucleus (216/399: 54.14%), cytoplasm (81/399: 20.3%) and chloroplast (70/399: 17.54%) (Table S1). Only a few proteins were located in the extracellular space (NtWD40-218 and NtWD40-339), vacuoles (NtWD40-251 and NtWD40-352) and peroxisomes (NtWD40-014 and NtWD40-151). The lengths and physicochemical properties varied greatly among the tobacco WD40 regulatory proteins. The lengths of the tobacco WD40 proteins ranged from 87 (NtWD40-050) to 2,378 (NtWD40-037) amino acids. The molecular weights (MWs) ranged from 9.871 (NtWD40-050) to 262.21 kDa (NtWD40-037), and the theoretical isoelectric points (pIs) ranged from 4.151 (NtWD40-067) to 10.427 (NtWD40-308) (Table S1).

### Phylogenetic, gene structure and conserved motif analysis

**Fig. 2:**
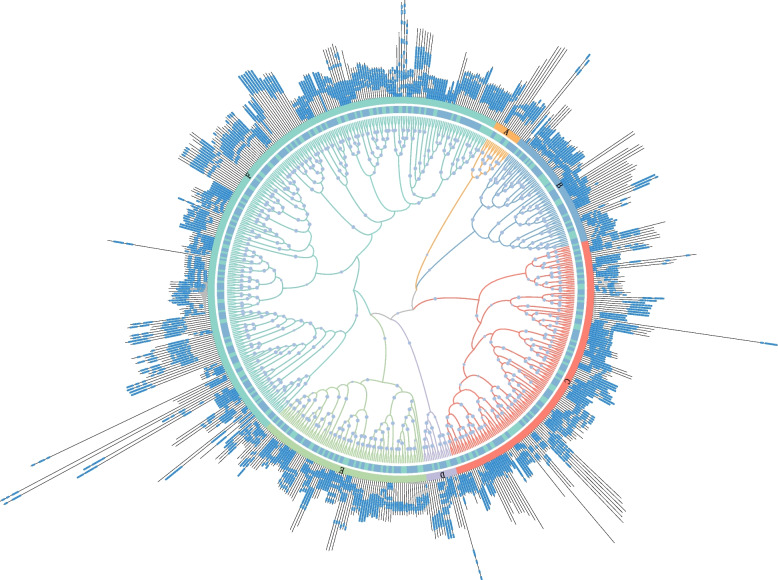
Phylogenetic classification of NtWD40 proteins. Different colors in outer circle denote clusters **A**–**F**. The blue color in inner circle represents tobacco and the green color in inner circle represents Arabidopsis

To further analyze the evolutionary relationships of the WD40 regulatory genes in tobacco, 399 tobacco WD40 proteins and 230 *Arabidopsis* WD40 proteins were used to construct a phylogenetic tree. The tobacco WD40 regulatory genes could be divided into 6 major distinct clusters (Clusters A–F), containing 25, 30, 59, 42, 104 and 139 proteins, respectively (Fig. 2; Table S1). Genes with similar structures were distributed in the same cluster. Figure 2 showed that the structures of proteins from between *Arabidopsis* and tobacco were similar. Specifically, NtWD40-389, TTG member named *NtTTG1*, was located in the F cluster (Fig. 2).

Interestingly, different NtWD40 clusters might vary significantly from each other (Fig. S1). For example, the protein lengths and molecular weights of the proteins in the F cluster were much greater than those of proteins in the other clusters. The WD40 genes in Cluster A trended to be more hydrophobicity than those in the other clusters. Clusters C and D had more nuclear‒localized members than those in the other clusters. To obtain further insights into WD40 evolution, we examined the exon‒intron structures of the NtWD40 genes (Table S1) and found that 27 NtWD40s had only one exon and no introns, 173 NtWD40s contained 2–9 exon, 171 NtWD40s contained 10–20 exons, and 28 NtWD40s contained more than 20 exons. Interestingly, the NtWD40 genes in Cluster B had more exons than those in the other clusters (Fig. S1). The 10 most conserved motifs for the tobacco WD40 regulatory genes were investigated using the MEME program and annotated using InterProScan (Table S2). Eight motifs (1, 2, 3, 4, 5, 6, 9 and 10) were annotated as WD40 repeats (Fig. S2A) and were present in most of the NtWD40s (99.7%, 99.7%, 90.2%, 94.7%, 78.4%, 80.2%, 77.7%, and 74.7%, respectively) (Fig. S2B), suggesting that these motifs had been preserved for a long period of time.

### Gene duplication events and collinearity analysis of the WD40 regulatory genes in tobacco

**Fig. 3:**
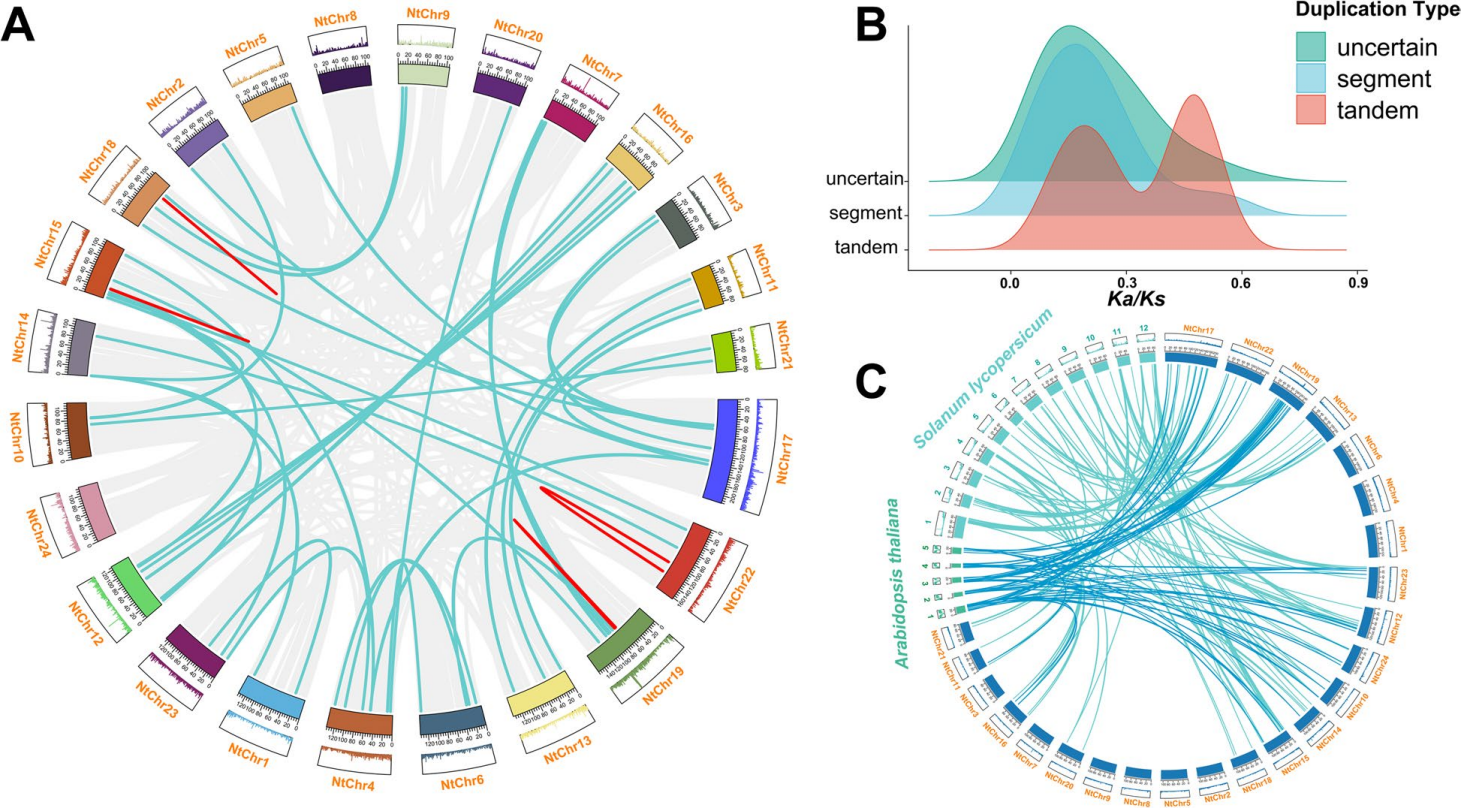
Duplication and collinearity analysis of NtWD40 genes. **A** Tandem and segmental duplicated gene pairs are linked by the red in same chromosome and cyan lines between chromosomes, respectively. **B** Ka/Ks distribution for different duplication events. **C** Micro-collinearity relationship between WD40 regulatory genes in tobacco and those in Arabidopsis and tomato

Since gene duplication was an important mechanism for the evolution of novel gene functions, different duplication events of NtWD40 regulatory genes were investigated in this study. A total of 206 duplication events involving 342 paralogs were detected, including 41 segmental duplications, 4 tandem duplications and 161 uncertain duplication events (Fig. 3A). The ratios of nonsynonymous (*Ka*) to synonymous (*Ks*) substitution rates were calculated to explore the evolutionary trajectory of duplicated tobacco WD40 regulatory genes (Table S3). *Ka/Ks* = 1 was regarded as neutral selection, *Ka/Ks* < 1 indicated purifying selection, and *Ka/Ks* > 1 referred to positive selection. Among the 206 duplicated events, with the exception of 9 uncertain events, the *Ka/Ks* ratios of all the analyzed duplication events were less than 1 (Fig. 3B), indicating the predominance of strong purifying selection on these genes throughout their evolutionary history. This suggested that functional differentiation following gene duplication was limited by purifying selection.

To further explore the evolutionary patterns of WD40 regulatory genes among different plants, two representative species, *Arabidopsis* and tomato, were selected for comparative collinearity analysis with tobacco. A total of 58 pairs of genes were collinear between tobacco and *Arabidopsis*, and 125 pairs were observed between tobacco and tomato (Fig. 3C). The number of collinear gene pairs between tobacco and tomato was much greater than that between tobacco and *Arabidopsis*. The number of WD40 genes with collinearity between the different plants clearly varied widely, suggesting that the expansion of WD40 genes might have occurred during Solanaceae differentiation.

### Cis-acting elements and regulatory networks associated with the NtWD40s

**Fig. 4:**
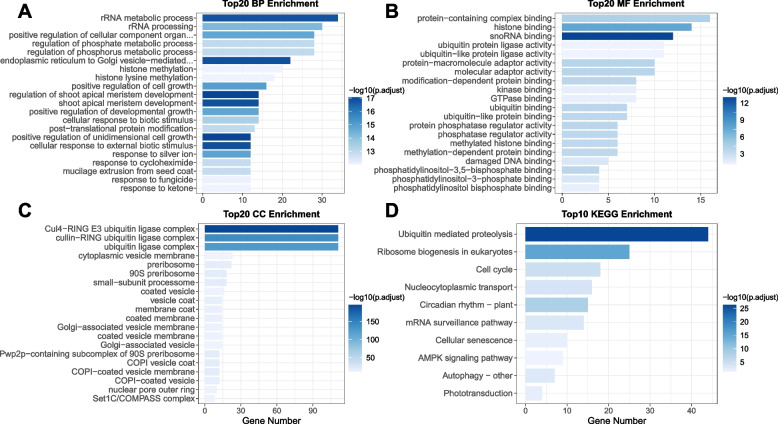
GO and KEGG enrichment for NtWD40 genes. **A** Bar plot for top 20 enriched biological process (BP) terms. **B** Bar plot for top 20 enriched molecular function (MF) terms. **C** Bar plot for top 20 enriched cellular component (CC) terms. **D** Bar plot for top 10 enriched KEGG pathways

To further understand the functions of the NtWD40s, GO and KEGG analyses were performed. The GO results revealed that the NtWD40 regulatory genes might play important roles in the development of different tissues, including shoot apical meristem development; the positive regulation of developmental growth; mucilage extrusion from the seed coat; and various stress responses, including cellular responses to biotic stimuli and cellular responses to external biotic stimuli (Fig. 4A, 4B and 4C). Similar results were also observed in the KEGG pathway analysis, including circadian rhythm-plant nucleocytoplasmic transport, cell cycle, and ubiquitin-mediated proteolysis (Fig. 4D; Table S4).

Considering that tobacco WD40s might participate in the development of different tissues and stress responses, we explored *cis*-acting elements (CREs) in the promoter regions of the NtWD40 genes to better understand the potential genetic regulatory mechanism involved. These identified CREs can be further divided into four types (Table S5): light-responsive (46.6%), phytohormone-responsive (28.3%), stress-responsive (9.3%) and development related elements (15.8%). The most abundant elements were light-responsive elements, including Box 4, G-Box, and the GT1 motif. ABRE, CGTCA-motif and TGACG-motif responsive elements accounted for the majority of phytohormone-responsive elements, with 657, 448, and 448, respectively. These large numbers of phytohormone-responsive elements indicated that hormone signals may regulate the expression of NtWD40 regulatory genes. Notably, all four CRE types were observed in the majority of WD40 genes (313/399:78.45%), indicating various regulatory roles for the NtWD40 genes (Fig. S3).

**Fig. 5:**
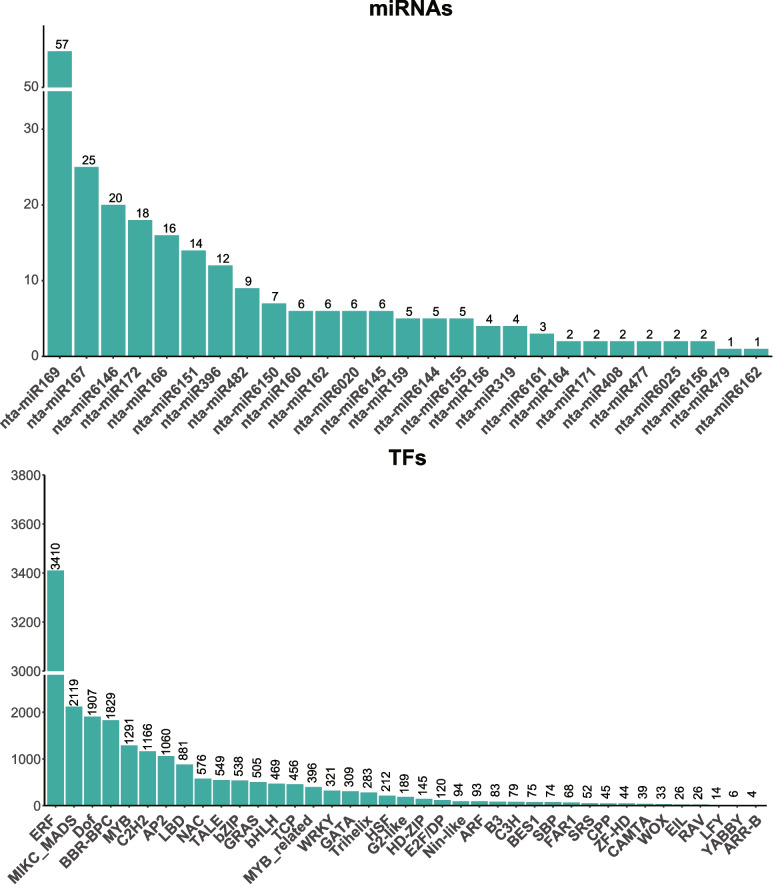
Visualization of miRNAs and transcription factor (TF) that could target NtWD40 genes

Due to the enrichment of numerous CREs in the promoter regions of the NtWD40 genes, we speculated that the corresponding transcription factors (TFs) may directly regulate the NtWD40 genes in tobacco. Hence, we investigated the relationship between TFs and NtWD40s using PlantTFDB. A total of 395 TF members from 39 families might play important roles in the regulation of NtWD40s (Fig. 5; Table S6). Among these TFs, ERF, MADS, Dof, MYB, C2HE, AP2, and bHLH were the most abundant. In addition, potential miRNA-binding sites for the NtWD40s were also explored using PsRNATarget. Overall, 75 miRNAs in 27 miRNA families might have potential regulatory relationships with NtWD40s (Fig. 5; Table S6). Most of the miRNAs had multiple target genes, of which the nta-miR159 and nta-miR162 families could target 5 and 3 NtWD40s, respectively. In contrast, several NtWD40s could be targeted by several miRNAs. For example, NtWD40-003 could be targeted by two miRNAs, nta-miR160 and nta-miR161, and NtWD40-012 could be targeted by three miRNAs, nta-miR408, nta-miR6146, and nta-miR6155. Hence, additional studies were needed to determine the regulatory relationship between NtWD40s and TFs/miRNAs.

### Tissue expression analysis of NtWD40 regulatory genes

**Fig. 6:**
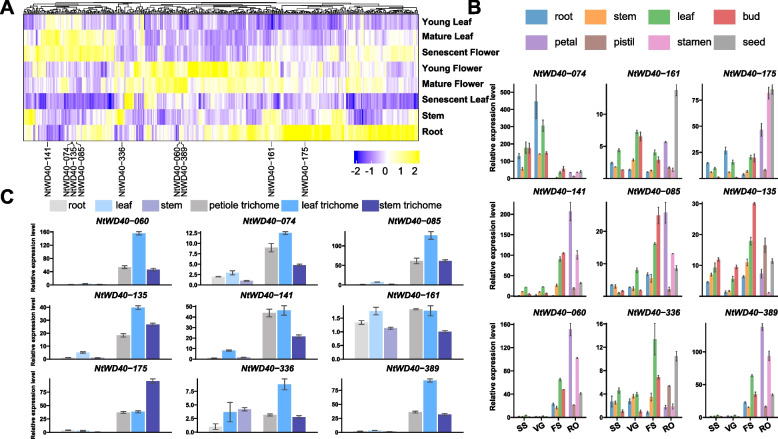
Expression profile of NtWD40 gene in different tissues. **A** Heatmap of the expression of NtWD40s in different tissues using public RNA-seq data, scaled by column. **B** qRT-PCR analysis of nine selected NtWD40s in different tissues at three developmental stages. Data are presented as mean ± SD. **C** qRT-PCR analysis of nine selected NtWD40s in trichome tissues. Data are presented as mean ± SD

To further study the potential function of the NtWD40 genes, the expression patterns of eight representative tissues were explored. With the exception of 12 NtWD40s that were not expressed or were weakly expressed in the eight tissues, the remaining 387 genes were expressed in at least one tissue (Table S7). Interestingly, numerous NtWD40 regulatory genes, particularly NtWD40-035, NtWD40-088, NtWD40-298, and NtWD40-395, exhibited high expression in the roots, suggesting that these genes might play crucial roles in root development (Fig. 6A). Similarly, several NtWD40 genes, such as NtWD40-291 and NtWD40-375, were highly expressed specifically at flowering stage. In addition, several NtWD40 genes might be expressed in different tissues at different developmental stages. For example, some NtWD40 genes, such as NtWD40-008, NtWD40-161, NtWD40-275, and NtWD40-359, were unexpressed or expressed at low levels in young leaves and senescent flowers, but were expressed at higher levels in other developmental stages, revealing the differing functions of the NtWD40 genes during leaf and flower development.

Nine NtWD40 genes (NtWD40-060, 074, 085, 135, 141, 161, 175, 336, and *NtTTG1*) with specific expression patterns in different tissues were randomly selected for further qRT‒PCR analysis, and the results were similar to those of the RNA‒seq analyses (Fig. 6B). NtWD40-060, NtTTG1, NtWD40-175, and NtWD40-141 were highly expressed at the flowering stage, especially in petals and stamens. In contrast, the expression of NtWD40-074 decreased significantly at the flowering stage. NtWD40-161 was highly expressed specifically in the seeds, NtWD40-336 was highly expressed in leaves at the flowering stage and in the seeds at the reproductive organ stage, and NtWD40-135 was highly expressed in the buds at the flowering stage. To further explore the expression patterns of the NtWD40s in trichomes, trichomes from the leaf, petiole and stem were explored among the different NtWD40s (Fig. 6C). The results showed that except for NtWD40-161, most of the NtWD40 genes examined, especially for NtTTG1, NtWD40-085, NtWD40-175, and NtWD40-060, were highly expressed in trichome tissues compared with expression in leaf and stem, indicating these NtWD40s might be involved in trichome development.

### Expression analysis of NtWD40 genes in response to various abiotic and biotic stresses

**Fig. 7:**
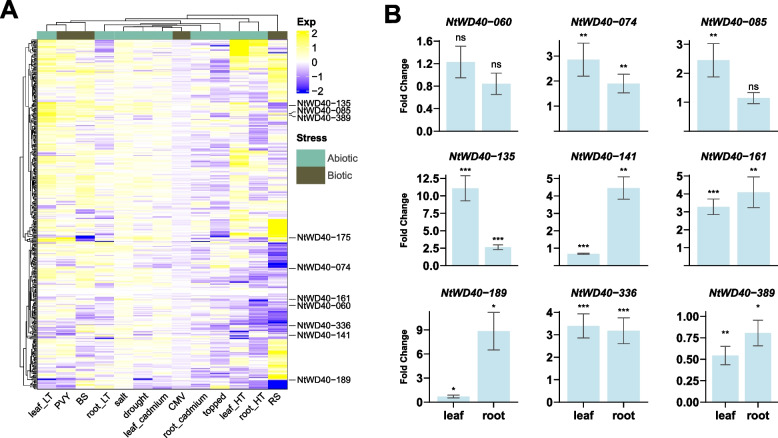
Expression profile of NtWD40 gene in various stress treatment. **A** Heatmap of the expression of NtWD40s under high temperature (HT), low temperature (LT), R. solanacearum (RS), black shank (BS), Cucumber Mosaic Virus (CMV), cadmium, salt, drought, Potato Virus Y (PVY) and topping treatment using public RNA-seq dataset. The expression change is indicated by the ratio of TPM value between the treatment and control (CK). **B** Fold change of the nine NtWD40s between expression of control and drought treatment. Note: Data are presented as mean ± SD, with one, two, and three asterisks denoting statistical significance at *p* < 0.05, *p* < 0.01, and *p* < 0.001, respectively

To further explore the regulatory role of NtWD40 in various stress treatments, the expression patterns of the NtWD40s were studied using publicly available transcriptome data. As shown in Fig. 7A, the NtWD40s exhibited distinct response patterns to the different stress treatments. The expression of most of the NtWD40 genes did not significantly change in the leaves of tobacco plants subjected to cucumber mosaic virus (CMV), salt or cadmium treatment (Table S8). Notably, the response patterns of the NtWD40 regulatory genes differed between leaf and root tissues under similar stresses, such as high temperature, low temperature, and cadmium treatment. For example, the majority of NtWD40 genes were upregulated in leaves under low temperature, while the opposite patterns were observed in roots, in which most of the genes were downregulated. Similarly, the expression of numerous NtWD40 genes significantly decreased in cadmium-treated tobacco root tissue but exhibited no change or were upregulated in the leaves, suggesting a tissue-specific response of NtWD40s under different stress treatments. In addition, different NtWD40s might exhibit opposite response patterns to various stress treatments. For example, the expression of several genes (such as NtWD40-161 and NtWD40-175) was significantly upregulated after salt treatment, while that of other genes, such as NtWD40-049 and NtWD40-074, was significantly downregulated. Under drought treatment, most NtWD40 genes, such as NtWD40-175, NtWD40-336, NtWD40-074, NtWD40-085, and NtWD40-135, were significantly upregulated, while some, such as NtWD40-141, NtWD40-189, and NtWD40-254, were significantly downregulated. Interestingly, NtWD40-212, which was not expressed in any tissues of the wild type, was significantly upregulated in both leaves and roots under both high and low temperatures, indicating its specific role in the temperature response.

To compare the roles of the NtWD40s among the different stress treatments, nine genes with distinct response patterns were selected for further investigation of their expression patterns via qRT‒PCR. Under drought treatment, NtWD40-074, 135, 161, and 336 were significantly upregulated in both leaf and root tissues (Fig. 7B). Similarly, NtWD40-141 and NtWD40-189 exhibited opposite expression patterns between leaf and root tissue, indicating tissue-specific responses to drought stress. In contrast, NtWD40-389 was significantly downregulated in both leaf and root tissues. Similarly, NtWD40-074, 085, 135, 161, and 336 were also upregulated under salt treatment (Fig. S4). Additionally, we observed gradually increasing expression patterns for these four genes in both root and leaf tissues under salt treatment, with the highest expression occurring at 8 h. NtWD40-389 presented opposite expression patterns between the leaf and root tissues, indicating tissue-specific responses to salt stress. In response to cold treatment, the expression of NtWD40-135/189 in leaves and roots significantly increased after 39 h and decreased after 89 h, but the NtWD40-389 (*NtTTG1*) only shows this phenomenon in leaves. (Fig. S5A). In contrast, the NtWD40-060/085 ratio gradually decreased under cold treatment. Interestingly, compared with the expression of other hormones, the expression of NtWD40-389 (*NtTTG1*) was significantly induced by IAA treatment, indicating its potential function in plant growth (Fig. S5B). In contrast, the expression of NtWD40-074 significantly decreased under all the hormone treatments except IAA. NtWD40-175 exhibited different response patterns to different hormones. When tobacco plants were infected with powdery mildew, most genes, such as NtWD40-060, 074, 085, 135, 141, 175, and 389, were upregulated, although NtWD40-189 and NtWD40-336 were not (Fig. S5C). In summary, the distinct expression patterns for NtWD40 genes indicated that they might be involved in different abiotic and biotic stress responses.

### Silencing of NtTTG1 enhanced drought tolerance of tobacco

**Fig. 8:**
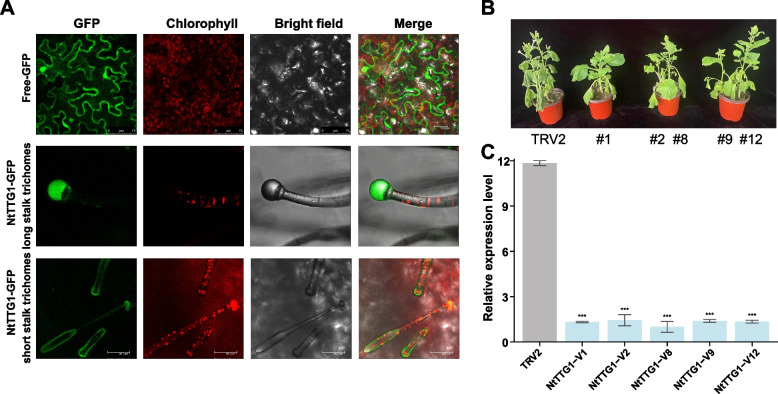
Subcellular localization of NtTTG1 and drought tolerance assessment in NtTTG1-silenced tobacco plants. **A** Subcellular localization of NtTTG1. The scale bars represent 20 μM. **B** Phenotype of NtTTG1-silenced tobacco plants after 5 days of drought treatment. **C** Relative expression of NtTTG1 detected by qRT-PCR before drought treatment. Note: Data are presented as mean ± SD, with one, two, and three asterisks denoting statistical significance at *p* < 0.05, *p* < 0.01, and *p* < 0.001, respectively

We demonstrated that one of the TTG proteins, NtWD40-389 (named *NtTTG1*), was highly expressed in trichome tissue and exhibited significant downregulation under drought stress and upregulation under IAA treatment. To further explore the influence of NtWD40s on tobacco drought tolerance, we constructed VIGS-silenced plants harboring *NtTTG1*. Subcellular localization analysis indicated that *NtTTG1* was preferentially localized in the nuclei of trichomes (Fig. 8A), which was consistent with the predicted results (Table S1) as well as findings in other species [42]. Compared with those of wild-type plants, the transcript abundance of *NtTTG1* was significantly lower in positively silenced plants (Fig. 8B and 8C). After five days of drought treatment, the wild-type plants exhibited more severe wilting, but the middle and upper leaves of the *NtTTG1*-silenced plants had spread out (Fig. 8B), indicating that silencing *NtTTG1* promoted the growth of the tobacco plants and enhanced their drought tolerance.

## Discussion

WD40 proteins have been reported to play important roles in plant development and various physiological processes [31, 39]. However, studies related to the WD40 genes in the tobacco family are rare. With the progress in producing high-quality tobacco genome data, it has become possible to investigate tobacco WD40 proteins at the genome-wide level. This study was the first comprehensive and systematic report on the characteristics of the NtWD40 gene family in tobacco. A total of 399 NtWD40 proteins were identified and divided into 6 major distinct clusters (Clusters A–F), containing 25, 30, 59, 42, 104 and 139 proteins (Fig. 2). The number of NtWD40 genes in tobacco (399) was greater than that in most other species, such as the dicotyledonous species *Arabidopsis* (230), cucumber (191), *Cucurbita maxima* (231), *sugar beet* (177), and the monocotyledonous species rice (200). However, the number of DEGs was lower than that in cotton (579) and wheat (743), which implied considerable expansion of the WD40 gene family in tobacco, cotton and wheat compared with that in other plants. Common wheat, which has a relatively large genome size (17 GB), indeed contained the highest number of WD40 regulatory genes. However, for plant species whose genome size was smaller than that of tobacco, such as terrestrial cotton (2.5 GB), there was a greater abundance of WD40 regulatory genes. These results suggested that there might be no correlation between genome size and the number of WD40-regulated genes. In plants, polyploidy and gene duplication are often the main triggers for the expansion of gene families. Tobacco is a heterotetraploid plant that resulted from interspecific hybridization between *N. tomentosiformis* (2*n* = 24) and *N. sylvestris* (2*n* = 24) nearly 200,000 years ago. Similarly, common wheat and terrestrial cotton experienced genome-wide tripling duplication (WGT) and genome-wide duplication events, respectively [43, 44]. Commonly, duplication and homologous recombination are the main drivers of gene family evolution, and changes in family size and gene family distribution are associated with tandem or fragment duplication [11, 29]. Gene family expansion occurs mainly through three modes: fragment duplication of multiple genes, tandem duplication of single genes, and genome-wide duplication [45]. A total of 206 duplication events were found, including 41 segmental duplications, 4 tandem duplications and 161 uncertain duplication events (Table S3). Fragment duplication was considered the main driver of NtWD40 evolution, as 19.9% (41) of the duplication were fragment events (Table S3). *Ka* and *Ks* analyses of the duplicated gene pairs suggested that both were subject to purifying selection, which is consistent with findings in studies of wheat and Qingke [15, 46].

*Cis*-regulatory elements (CREs) are important for regulating gene activity in various biological processes. In the present study, by analyzing the promoter regions of NtWD40s in tobacco, numerous CREs related to the plant stress response, hormone response, and growth were identified. Similar results were also found for the promoter regions of WD40 regulatory genes in other plant species, such as *Cucurbita maxima*, Qingke, and cotton [14, 16, 46]. Based on these *cis*-elements, we found that 394 TF members from 39 families might play important roles in the regulation of NtWD40s. Many TF families implicated in stress responses, including WRKY, MYB, NAC, and bHLH, have been identified. The interactions between WD40 proteins and MYB and bHLH transcription factors have been widely investigated in flavonoid biosynthesis [42, 46]. In strawberry fruits, FaMYB5/FaMYB10-FaEGL3 (bHLH)-FaLWD1/FaLWD1-like (WD40) directly targeted the promoters of F3'H, LAR, and AHA10, thus committing to flavonoid accumulation. Similarly, in *Raphanus sativus*, *RsTTG1*, a WD40 protein, could interact with the bHLH transcription factor *RsTT8* to regulate anthocyanin and proanthocyanidin biosynthesis [41]. Additionally, the TTG1-bHLH-MYB complex might be directly involved in the regulation of root growth and trichome patterning in *Arabidopsis* leaves [47]. Based on the results of *cis*-element analysis in our study, a WD40-bHLH-MYB interaction model was constructed. Finally, only two NtWD40 genes (NtWD40-060 and NtWD40-389, named *NtTTG1*) were identified in the WD40-bHLH-MYB model; these genes could also be regulated by three bHLH family members (Fig. S6). Moreover, several MYB and bHLH families could interact with each other through corresponding *cis*-responsive elements, in line with the findings of previous studies [48]. However, no interaction was detected between the WD40 and MYB families. Hence, we speculated that the bHLH families might serve as intermediate bridges for the WD40 and MYB families, but additional studies were needed to validate this hypothesis.

In addition, to elucidate the functions and regulatory mechanisms of miRNAs and WD40s, we predicted miRNAs that might target tobacco WD40 genes. Twenty-seven miRNA families, consisting of 75 miRNAs, may have regulatory relationships with NtWD40s (Fig. 5; Table S6). Previous studies have revealed that moderate miR156 transcript level were sufficient to enhance drought resilience in alfalfa by silencing SPL13 and increasing WD40-1 expression, whereas higher miR156 transcript levels resulted in drought susceptibility [49]. Similarly, our results also indicated that NtWD40-379, which was a guanine nucleotide-binding protein subunit beta-like protein (G-protein β), contained binding sites for nta-miR156. Previous studies have proved that plant G-protein β subunits could positively regulate drought tolerance by increasing the detoxification of ROS [50]. Hence, we speculated that NtWD40-379, regulated by nta-miR156, might also play important roles in the drought tolerance of tobacco plants.

The analysis of tissue expression patterns in tobacco showed that many WD40 genes were expressed in distinct tissue expression patterns. A large number of WD40 regulatory genes were highly expressed in flower tissue (Fig. 6A), suggesting their specific roles in flower development. Chen et al. reported that convergent selection of a WD40 protein enhanced grain yield in maize and rice [51]. The WD40 domain protein GORI was an integrative scaffold that was needed for pollen tube growth in rice [32]. Our qRT‒PCR results also indicated that many genes were highly expressed at the flowering stage, suggesting that these genes might be involved in flower growth and seed development. For example, NtWD40-175, a JINGUBANG protein, was a homolog of GORI and was highly expressed at the flowering stage in our study (Fig. 6B).

Moreover, by analyzing plant response patterns under different stress treatments, we found that the expression of some NtWD40s did not significantly change in response to stress (Fig. 7A), suggesting that these genes may respond to plant stress through other pathways. It was worth noting that the same gene type may exhibit varying stress response patterns. For instance, NtWD40-024 exhibited robust responses to the biotic stresses *R. solanacearum* (RS) and black shank (BS) but displayed insensitivity to CMV or PVY infection (Fig. 7A). However, further investigations were needed to determine the disease response mechanisms of WD40 regulatory genes in tobacco. Specifically, we focused on the response of tobacco NtWD40s to drought and salt stresses. For the randomly selected genes, many genes, such as NtWD40-074 and NtWD40-389, exhibited opposite expression patterns under drought and salt treatments, indicating that different NtWD40s may have different mechanisms for these two stresses. Silencing of NtWD40-389 (*NtTTG1*) also confirmed its important regulatory role in drought stress (Fig. 8). In summary, the expression patterns of the NtWD40s under growth, development, and stress conditions were diverse, indicating their functional diversity. However, additional studies were needed to explore the detailed mechanisms of candidate NtWD40s in tobacco plants.

## Conclusion

The present study provides the first comprehensive and systematic identification and exploration of the WD40 gene family in tobacco. A total of 399 WD40 genes were identified and further classified into 6 clusters. The gene structure, conserved domains, and motifs of the NtWD40 genes were subsequently evaluated. Moreover, segmental duplication was found to be the major driver of the expansion of tobacco WD40 regulatory genes. Many NtWD40 genes were demonstrated to be expressed in a tissue-specific manner and to be involved in various biotic and abiotic stresses. Notably, *NtTTG1*-silenced tobacco plants exhibited significantly enhanced drought tolerance. These results could be useful for enhancing tobacco resistance to different stresses. Our study provided a valuable basis for further functional verification of WD40s in tobacco.

## Methods

### Identification and characterization of WD40 regulatory genes in tobacco

To identify all members of WD40 regulatory genes in tobacco, HMMER (v3.3.2) was employed using hidden markov model (HMM) based on the WD40 conserved domain (PF00400). All candidate sequences were confirmed using the Simple Modular Architecture Research Tool (SMART) (http://smart.embl-heidelberg.de/). TBtools was used to calculate the isoelectric points and molecular weights of tobacco WD40 proteins [52]. WoLF PSORT Online software (https://wolfpsort.hgc.jp/)was used to investigate the subcellular localization of tobacco WD40 proteins [53].

### Phylogenetic classification, gene structures and conserved motifs analysis

The FastTre software was used to construct the maximum likelihood tree of the tobacco WD40 proteins, with a bootstrap value of 1000, and visualized by ITOL (https://itol.embl.de/) [54, 55]. The detailed protein sequences were listed in Table S9. To reveal the exon–intron organization of the WD40 genes in tobacco, TBtools software was used to determine the gene structures of each WD40 gene [52]. To analyze the conserved motifs in WD40 regulatory genes, MEME software was employed with maximum number of motifs of 10 [56]. Subsequently, InterProScan was used to annotate these identified motifs [57].

### The chromosomal distributions, gene duplication and collinearity analysis

All tobacco WD40 regulatory genes were mapped to their corresponding chromosomes or scaffolds. Then, the origin, loss and expansion of WD40 regulatory genes in tobacco were inferred through phylogenetic tree. The duplication events of the tobacco WD40 genes were analyzed by BLAST (v2.13.0, e-value = 1e − 5), based on the similarity of amino acid sequences. Gene pairs with the similarity greater than 75% were considered as duplicated [58]. Gene pairs located on the same chromosome or scaffold are defined as tandem repeats, while those located on different chromosomes are defined as segmental repeats, and the rest are defined as uncertain. TBtools was used to calculate the non-synonymous (*Ka*) and synonymous (*Ks*) substitution rates of the duplicated events, and selective pressure was evaluated using the *Ka*/*Ks* ratio [52]. Collinearity analysis of WD40 genes was performed by TBtools and MCScanX between different species [59].

### Enrichment analysis, promoter analysis and interaction network prediction

To explore the function of the tobacco WD40 genes, all protein sequences were aligned to the emapper database (v5.0.2), with the species range set to Viridiplantae [60]. Subsequently, the R package ClusterProfiler was used for Gene Ontology (GO) and Kyoto Encyclopedia of Genes and Genomes (KEGG) enrichment analysis of tobacco WD40 genes [61–64]. For *cis*-acting element analysis, 2,000 bp upstream sequences from the transcription start site were extracted and submitted to the PlantCare database [65]. Mature miRNA sequences were downloaded from the miRBase database, and the psRNATarget database was used to search the regulatory relationship between miRNAs and tobacco WD40 gene [66, 67]. The regulatory relationship between tobacco WD40 genes and transcription factors (TFs) was retrieved from the Plant Transcription Factor Database PlantTFDB (http://planttfdb.cbi.pku.edu.cn) by searching for promoter sequences.

### Expression profiles based on transcriptome data

To investigate tissue-specific expression patterns of WD40 regulatory genes, transcriptome data from eight representative tobacco tissues (root, stem, young leaf, mature leaf, senescent leaf, immature flower, mature flower, and senescent flower) were utilized. To explore the response of WD40 regulatory genes to different biotic and abiotic stresses, tobacco transcriptome samples subjected to various biotic stresses such as Potato Virus Y (PVY), black shank (BS), Cucumber mosaic virus (CMV), and *Ralstonia solanacearum* (RS), as well as abiotic stresses including high/low temperature, drought, salt, cadmium, and topping were obtained from the NCBI SRA database. The accession numbers for all the transcriptome data utilized in this study could be found in Table S10. The Salmon software (v1.10.1) was applied to perform quantification on all clean read [68], the R package ‘tximport’ (https://github.com/mikelove/tximport) was used to calculate the transcripts per kilobase million (TPM) values, and TBtools software was used for visualization of expression profiling [52].

### Plant materials and stress treatment

The cultivated tobacco variety K326 was used to explore the expression of WD40 genes in various tissues and different stresses. Seedlings were cultured in plastic pots with a photoperiod of 16 h at 28 °C during the day and 23 °C at night. Root, stem, leaf, bud tissues at seedling stage (SS), vigorous growth stage (VG), and flowering stage (FS), as well as petal, stamen, pistil and seed samples at reproductive organs (RO) were sampled. To further investigate the expression of tobacco WD40 genes in trichome, trichome samples from leaf, petiole and stem were also collected. For salinity stress, 28 d old seedlings were treated with 400 mM NaCl for 3 h, 8 h, 12 h and 24 h, respectively. Same stage seedlings were also treated with plant hormones for 6 h, including ABA (10 μ M), IAA (10 μ M), Salicylic acid (SA) (10 μ M) and jasmonic acid (JA) (50 μ M and 100 μ M) [69, 70]. In addition, drought treatments were carried out by not watering for 7 days. Untreated seedlings were regarded as controls. All samples were independently collected with three independent replicates.

### RNA isolation and relative expression analysis

Total RNA was extracted using SuperPure Plantpoly RNA Kit (Gene Answer, Beijing, China) and DNA contamination was eliminated with RNase-free DNase I (Gene Answer). First-strand cDNA synthesis was performed using 1 μg of total RNA as a template by using reverse transcription kit (Trans, Beijing, China), and diluted to a concentration of 50 ng/µL. Real-Time Quantitative Reverse Transcription PCR (qRT-PCR) of tobacco WD40 genes was performed with SYBR Green kit (Trans, Beijing, China) with a reaction system of 20 μL. The PCR procedure used a two-step method consisting of an initial step of 95 °C for 30 s, followed by 40 cycles of 95 °C for 10 s and 60 °C for 30 s. The relative gene expression was calculated by 2^−∆∆Ct^ method and GAPDH was used as the internal reference gene. All qRT-PCR specific primers were listed in Table S11. Three independent biological replicates were conducted for qRT-PCR analysis.

### ***Subcellular localization analysis***

To verify the subcellular localization of WD40 genes in tobacco, primers were designed for segment-length coding sequences (CDS) of NtWD40-389 (which named as *NtTTG1* for its particularly high sequence similarity with *TTG1* gene in *Arabidopsis* and other plants). The subcellular localization vector *NtTTG1*-PC1300s-GFP was constructed by homologous recombination (Fig. S7A). Generally, the plasmids (*NtTTG1*-PC1300s-GFP and PC1300s-GFP) were transformed into Agrobacterium GV3101, and positive colonies were verified and expanded to OD value 1 at 28 °C. These colonies were re-suspended with MgCl_2_ + As + MES. After 3 h in darkness, they were injected into leaves of *Nicotiana benthamiana*. After injection, tobacco plants were cultivated in darkness for 1 d, and in normal condition for 1 d. Finally, GFP signals were detected using a confocal microscopy system (Nikon C2-ER, Japan).

### Functional assessment of NtTTG1 by virus-induced gene silencing

Wild-type *N. benthamiana* plants were cultivated in a climate-controlled chamber at a temperature ranging from 20–22 °C and exposed to a light period of 16 h. The virus-induced gene silencing (VIGS) vector *NtTTG1*-pYY13 was constructed similar with subcellular localization analysis (Fig. S7B). The plasmid (*NtTTG1*-pYY13) was transformed into Agrobacterium GV3101, and positive colonies were verified and expanded to OD value 1 at 28 °C. In addition, TRV1, TRV2 and PDS were also expanded. After these colonies re-suspended with MgCl_2_ + As + MES and kept in darkness for 3 h, *NtTTG1*-PYY13, TRV2 and PDS were mixed with TRV1 and injected into *N. benthamiana* leaves, respectively. After in darkness for 1 d, the injected plants were cultivated in a well-regulated growth chamber for 14 d. When PDS-silenced plants showed a severe phenotype with complete photo bleaching in all newly emerging leaves, qRT-PCR analysis was performed on uninfected leaves for each group. Subsequently, silenced plants were subjected to drought treatment, and corresponding phenotype were observed.

### Supplementary Information

**Additional file 1.**

## Data Availability

All data generated or analyzed in this study are included in the materials and methods section of this article.
