# Supplementary material for: Genome-wide identification and analysis of WD40 proteins reveal that NtTTG1 enhances drought tolerance in tobacco (Nicotiana tabacum)
Source: BMC Genomics. 2024 Feb 2;25:133. doi: 10.1186/s12864-024-10022-w (PMC10835901; doi:10.1186/s12864-024-10022-w)
Supplement: Supplementary file 1 — Additional file 1. [file 12864_2024_10022_MOESM1_ESM.zip › supplementary-revised/supplementary figures _revised.docx]

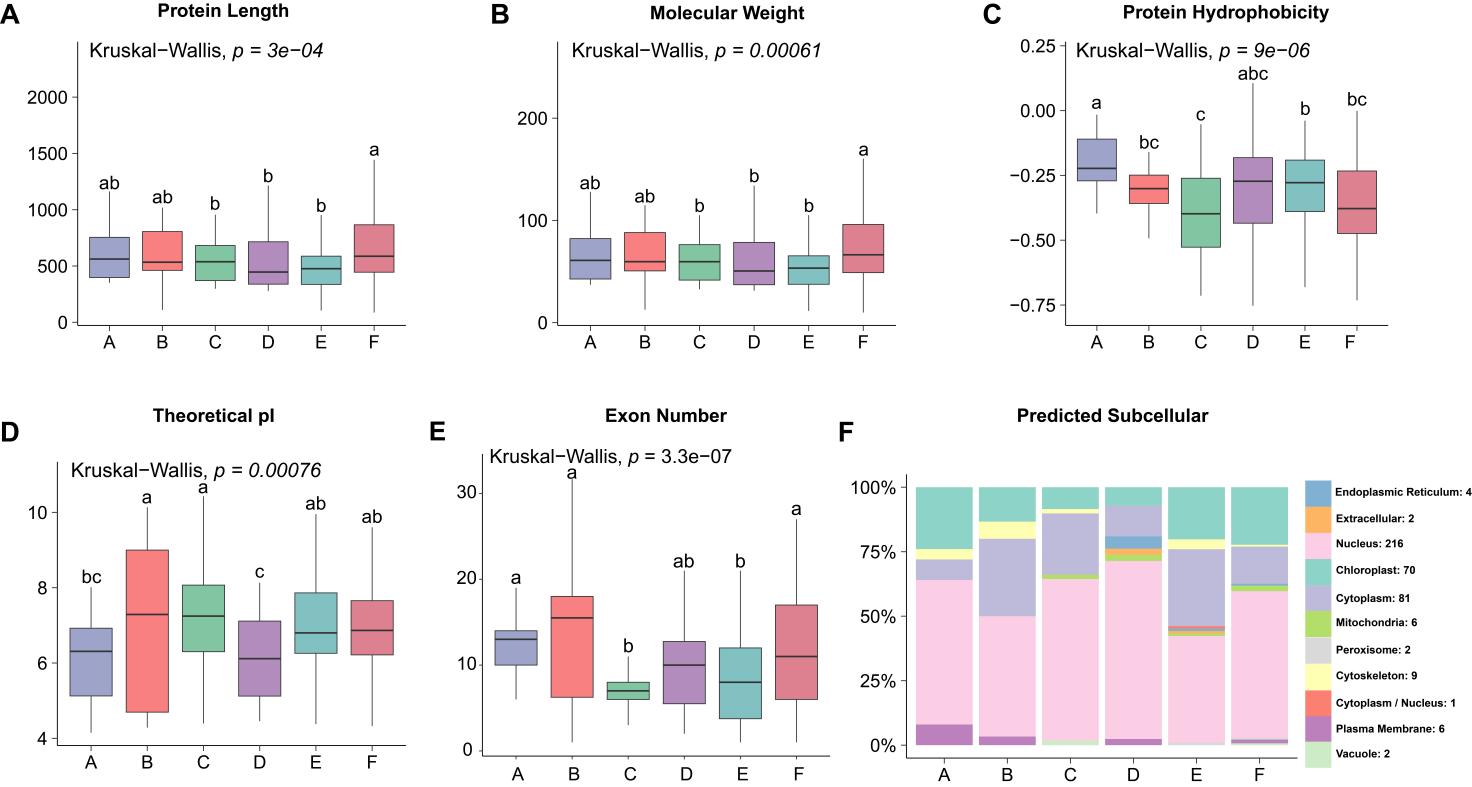


**Figure S1**. Statistical analysis for (A) protein length, (B) molecular weight, (C) protein hydrophobicity, (D) theoretical pI, (E) exon number, (F) predicted subcellular location. The P values in A – E were analyzed by ‘*t-test*’.


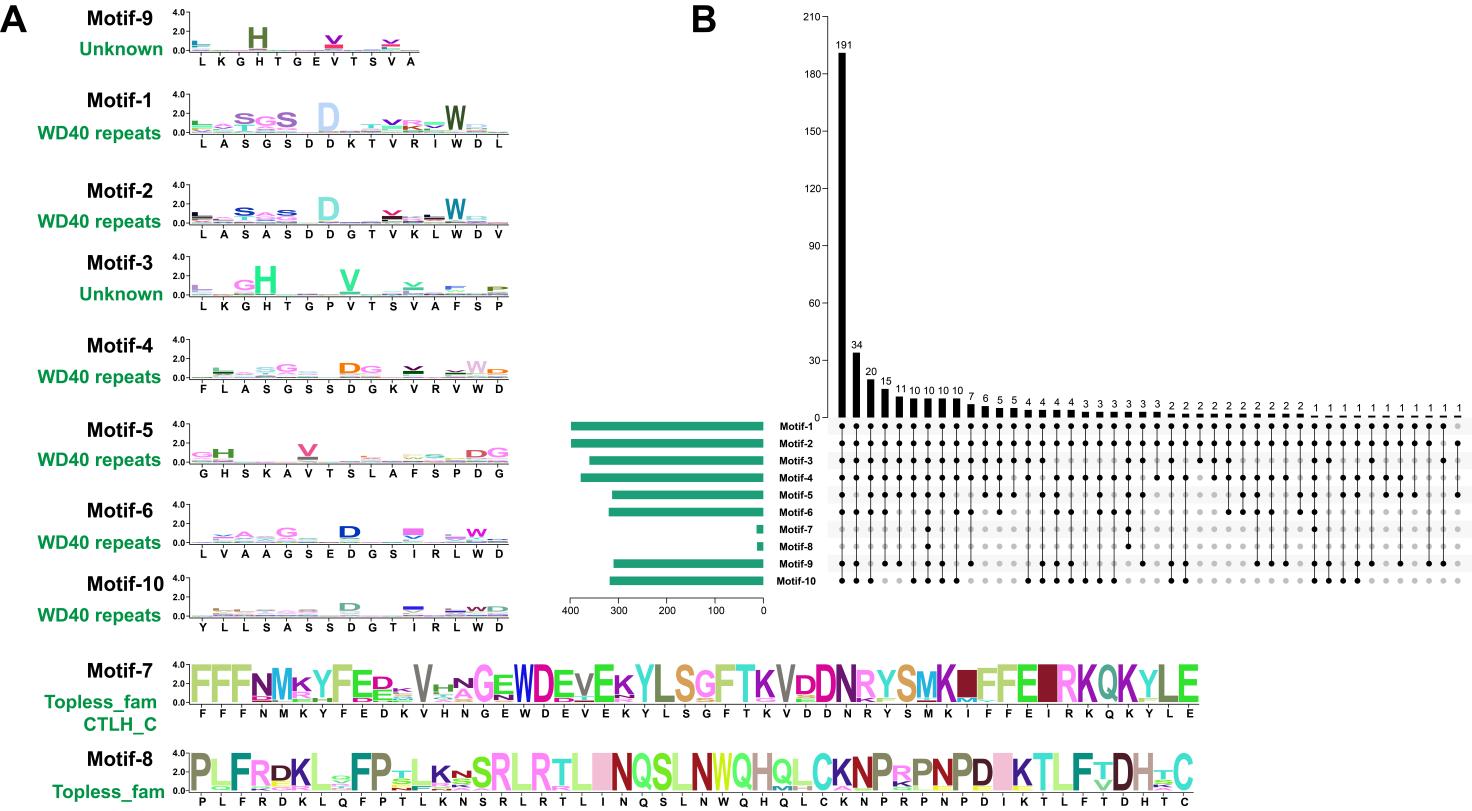


**Figure S2**. Visualization of top 10 motifs for NtWD40 proteins. (A) Sequence logo for top 10 motifs. (B) Upset graph for number of genes containing these top 10 motifs.


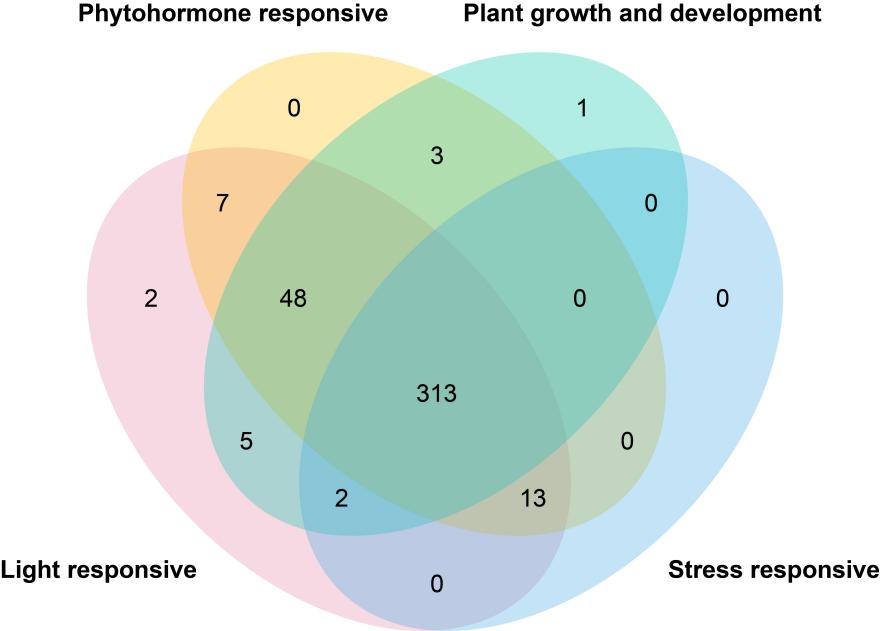


**Figure S3**. Venn graph of four types of cis-responsive elements.


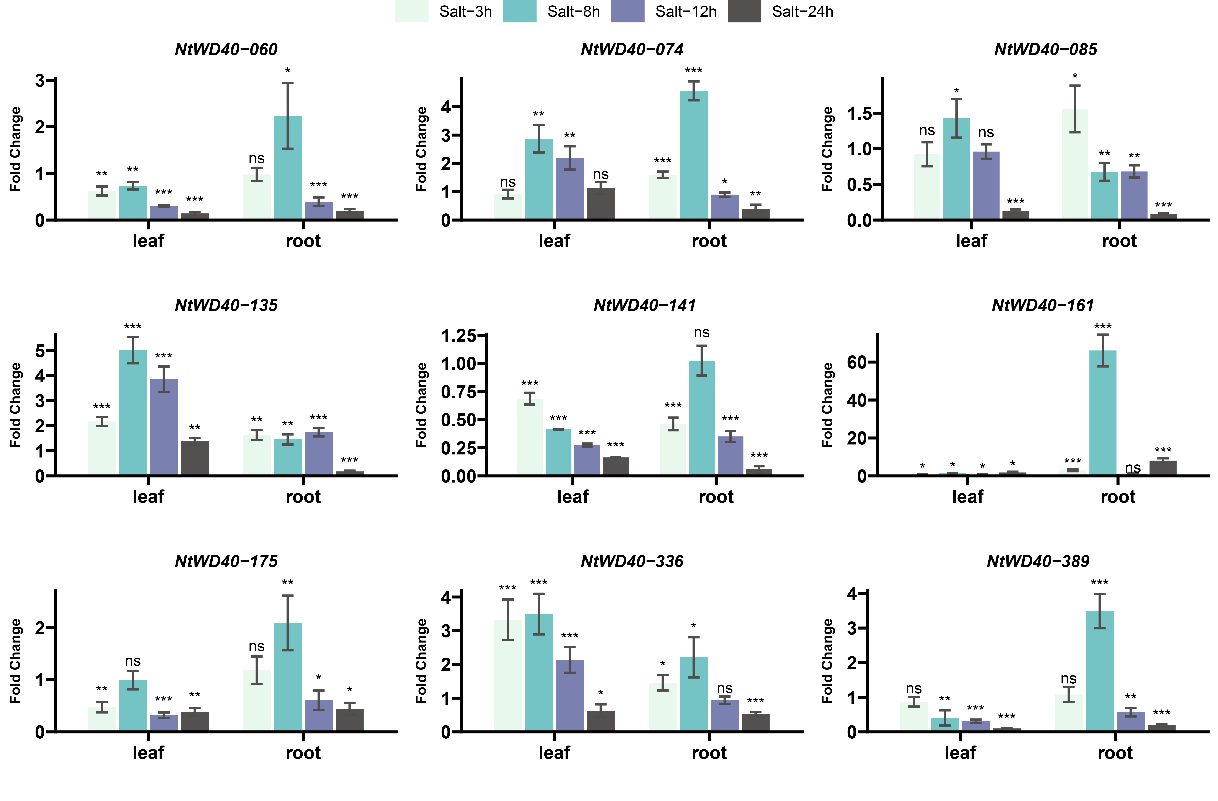


**Figure S4**. Fold change for the selected NtWD40 genes between expression of control and salt treatment. Note: Data are presented as mean ± SD, with one, two, and three asterisks denoting statistical significance at p < 0.05, p < 0.01, and p < 0.001, respectively.


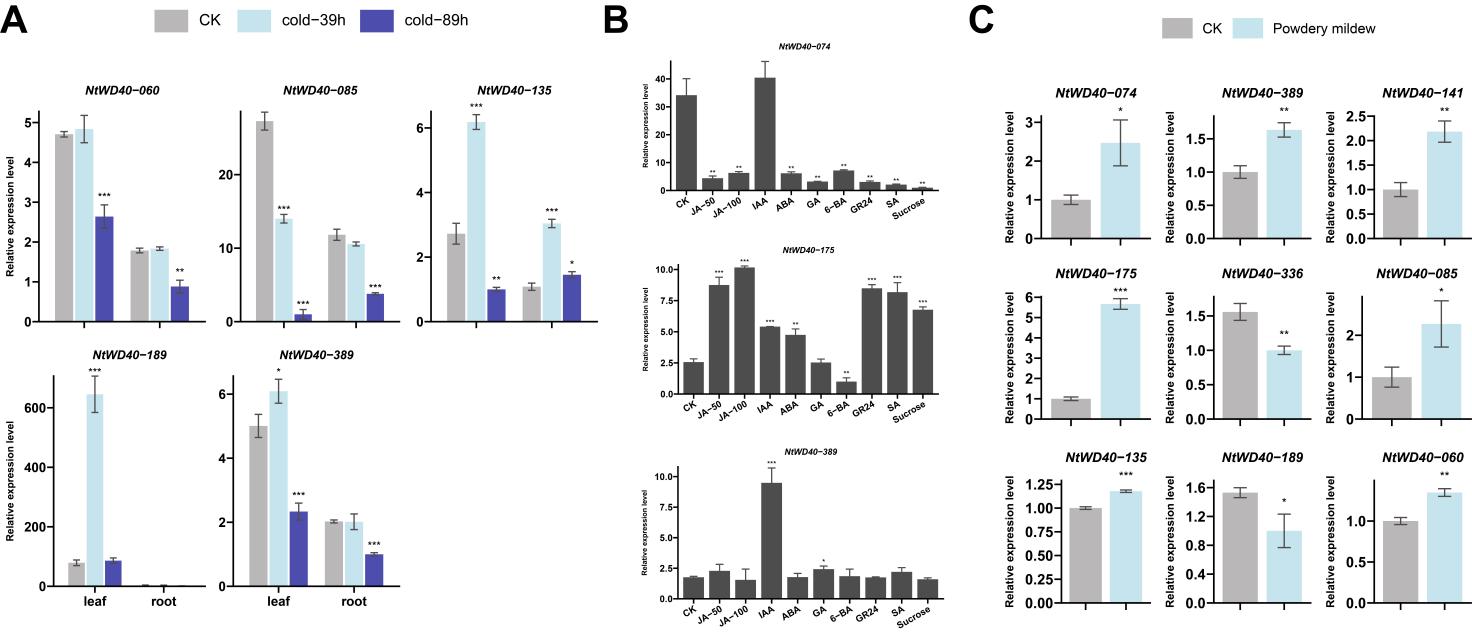


**Figure S5**. Expression profile of selected NtWD40 genes under cold (A), hormone (B), and powdery mildew (C) treatment. Note: Data are presented as mean ± SD, with one, two, and three asterisks denoting statistical significance at p < 0.05, p < 0.01, and p < 0.001, respectively.


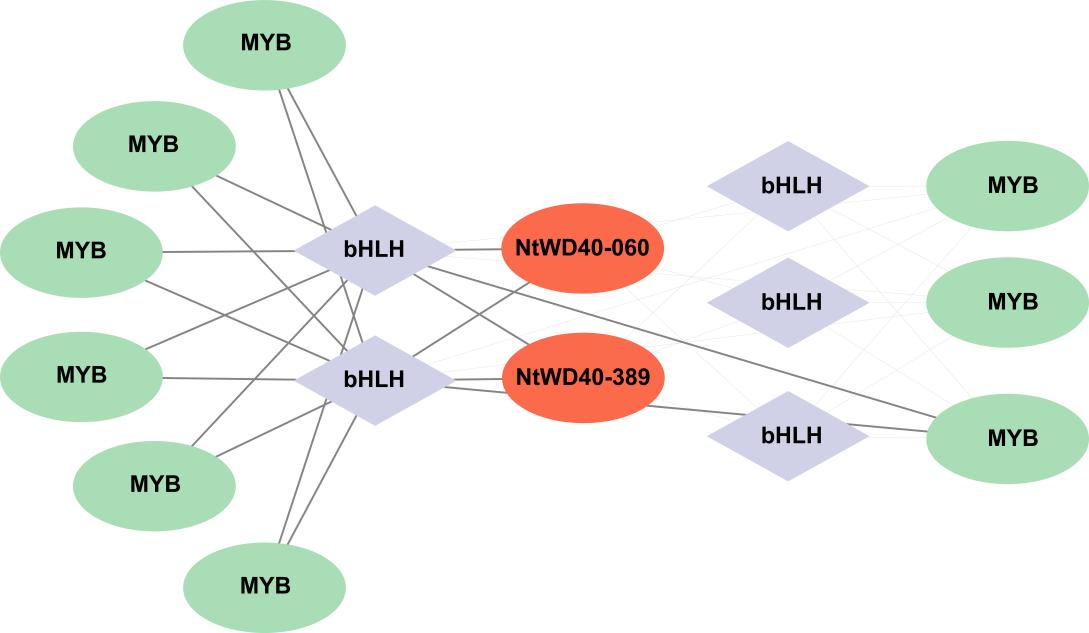


**Figure S6**. Potential WD40-bHLH-MYB regulation network of NtWD40 regulatory genes in tobacco.


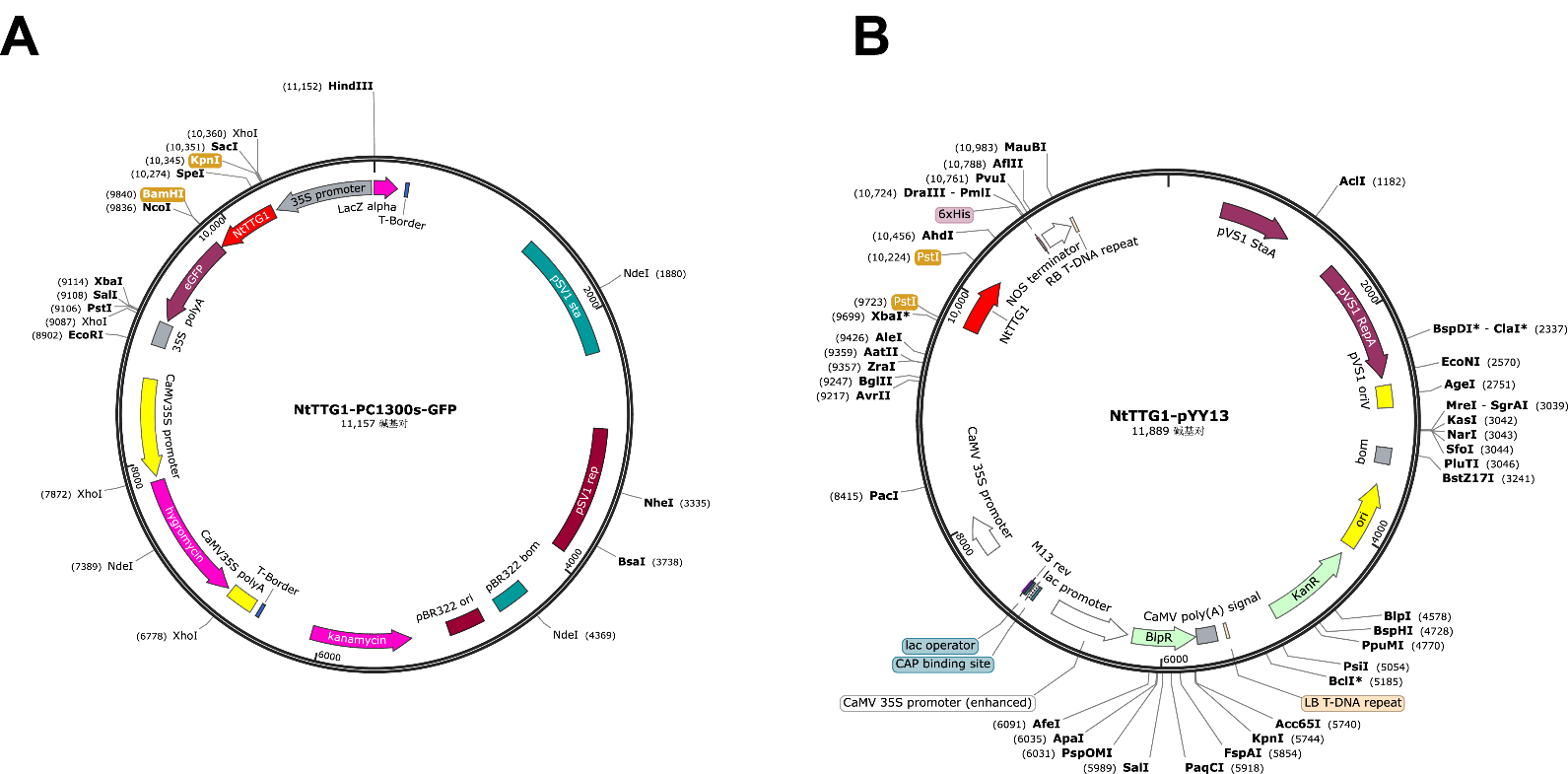


**Figure S7**. Constructed Subcellular localization vector NtTTG1-PC1300s-GFP (A), and VIGS vector NtTTG1-pYY13 (B).
